# Treatment of diaphyseal forearm defects caused by infection using Ilizarov segmental bone transport technique

**DOI:** 10.1186/s12891-020-03896-w

**Published:** 2021-01-07

**Authors:** Yanshi Liu, Maimaiaili Yushan, Zhenhui Liu, Jialin Liu, Chuang Ma, Aihemaitijiang Yusufu

**Affiliations:** 1 Department of Microrepair and Reconstruction, The First Affiliated Hospital of Xinjiang Medical University, Urumqi, Xinjiang China; 2 Department of Prosthodontics, The First Affiliated Hospital of Xinjiang Medical University, Urumqi, Xinjiang China

**Keywords:** Bone defect, Bone transport, External fixation, Forearm

## Abstract

**Background:**

The Ilizarov segmental bone transport technique can be applied in the reconstruction of the bone defects with less invasive fashion and more versatility compared to other methods, while most studies were focused on the lower extremity. The purpose of this study was to evaluate the effectiveness of the Ilizarov segmental bone transport technique in the treatment of diaphyseal forearm bone defects caused by infection.

**Methods:**

This study included 12 patients with diaphyseal forearm bone defects caused by infection, who underwent bone transport procedures using the monolateral external fixator at our institution from January 2010 to January 2018, including 10 males and 2 females with a mean age of 39 years (range 23–57 years). Patient’s demographic data and clinical outcomes at least two years follow-up after removing the external fixator were collected and retrospectively analyzed. The functional results were evaluated by the questionnaire of Disability of Arm, Shoulder and Hand (DASH) and the modified Mayo wrist score (MWS) at the final follow-up.

**Results:**

There were 10 radii and 2 ulnae bone transport procedures collected. The average defect size was 5.1 cm (4-6.5 cm). All patients were successfully followed up with a mean period of 28.2 months (24 to 36 months) and achieved infection-free union. There was no recurrence of infection observed. The mean external fixation time was 232.6 days (182 to 276 days), and the mean external fixation index was 46.3 days/cm (40.9 to 61.8 days/cm). The mean DASH score was 30.6(18 to 49) preoperative, while 13.8 (5 to 26) at the final follow-up. The average modified MWS improved from 68.8 (55 to 80) pre-operatively to 83.8 (65 to 90) at the final follow-up. All the differences between the preoperative and final scores were statistically significant (p < 0.05). Almost all the patients achieved satisfactory clinical outcomes and were able to perform activities of daily living.

**Conclusions:**

Ilizarov segmental bone transport technique is an alternative and effective method for the treatment of diaphyseal forearm bone defects caused by infection, and this method acquired satisfactory clinical outcomes.

## Background

Bone defects in the diaphyseal forearm may occur due to high-energy injury, the removal of contaminated and devascularized bony fragments in open fractures, resection of a bone tumor, or radical debridement of infected nonunion [1, 2]. The management of bone defects in the diaphyseal forearm is a real challenge for the restoration of the biomechanics of the elbow and wrist due to the close proximity of vital neurovascular structures and the necessity to maintain supination, pronation, and range of motion of the adjacent joints [3]. The different technical options for the reconstruction of diaphyseal bone defects are shortening, nonvascularized autografts, vascularized autografts, allografts, bone substitutes, and induced membrane technique [4–11], but the subsequent results are not completely satisfying. Another option is bone transport with distraction osteogenesis [12–14]. The variety of reconstruction methods reflects the complexity in achieving healing in the gap left by the bone defects.

The Ilizarov technique has been used for the management of high-energy complex fractures, bone nonunion, limb shortening, limb deformities, joint contractures, and bone defects over years [15–19]. For bone defects, the Ilizarov segmental bone transport technique can be applied in the reconstruction of any length theoretically with less invasive fashion and more versatility compared to other methods, especially for massive bone defects > 6 cm. Compared with the lower limbs, the upper limbs require more function than weight-bearing. Most studies about applying the Ilizarov technique in the treatment of bone defects were focused on the lower extremity [12–14, 20–24], while there were few published data reported in the upper extremity due to the complexity of the bone transport procedure and for fear of functional loss [2, 3, 25–28]. The purpose of our study was to evaluate the effectiveness of the Ilizarov segmental bone transport technique in the treatment of diaphyseal forearm bone defects caused by infection.

## Methods

This retrospective study included 12 patients with diaphyseal forearm bone defects caused by infection, who underwent bone transport procedures using the monolateral external fixator (Limb Reconstruction System, LRS, Orthofix, Verona, Italy) at our institution from January 2010 to January 2018. There were 10 males and 2 females with a mean age of 39 years (range 23–57 years). Patients older than 18 years with bone defects larger than 4 cm in the diaphyseal forearm were included in the present study. We excluded patients with pathological fracture, bilateral fracture, fracture associated with vascular and nerve injury, age older than 65 years, poor compliance, and any other illness that can affect bone healing (including diabetes, hypertension, osteoporosis, etc.). Informed consent was obtained from all patients for their data to be recorded and published in our study. This study was approved by the Ethical Committee of our institution.

The bone transport procedure involved 10 radii (left in 6, right in 4) and 2 ulnae (left in 2). The etiology of bone defect included osteomyelitis (primary or posttraumatic osteomyelitis) in 9 (7 radii and 2 ulnae) and infected nonunion in 3 (3 radii). For the location of bone defect, proximal 1/3 of the diaphysis in 3 cases (2 in radii, 1 in ulna), middle 1/3 in 7 cases (6 in radii, 1 in ulna), and distal 1/3 in 2 cases (2 in radii). The mean number of operations before presenting to our institution was 2.2 (0–4 operations). The average defect size was 5.1 cm (4-6.5 cm) measured intraoperatively after radical debridement. There were 7 limbs in an active infected state with sinus and drainage. Samples obtained from drainage or deep tissue at the infected site were cultured and conducted antibiotic susceptibility tests in all patients. The results showed 8 patients infected with Staphylococcus aureus, 2 patients with Pseudomonas aeruginosa, Methicillin-resistant Staphylococcus aureus and Escherichia coli infected one patient respectively. (More details are shown in Table 1)

**Table 1:** Details of the patients

Case	Sex	Age (years)	Disease	Location	Defect size(cm)	Previous operation time(s)	Infecting organism
1	Female	41	O	L, Radius, middle	5	2	SA
2	Female	34	IN	L, Radius, distal	4	3	SA
3	Male	43	O	R, Radius, middle	4.5	1	PA
4	Male	25	O	L, Radius, middle	4.5	0	SA
5	Male	38	IN	L, Radius, proximal	4	4	SA
6	Male	46	O	R, Radius, middle	5	3	MRSA
7	Male	57	O	R, Radius, distal	4.5	2	SA
8	Male	43	IN	L, Radius, middle	6	2	SA
9	Male	51	O	L, Radius, middle	5.5	3	E. coli
10	Male	41	O	R, Radius, proximal	6.5	3	SA
11	Male	23	O	L, Ulna, proximal	6	1	SA
12	Male	31	O	L, Ulna, middle	5.5	2	PA

*O* Osteomyelitis; *IN* Infected nonunion; *SA* Staphylococcus aureus; *MRSA* Methicillin-resistant Staphylococcus aureus; *PA* Pseudomonas aeruginosa; *E. coli* Escherichia coli; *L* Left; *R* Right

### Surgical technique

#### Stage I( eradication of infection)

The patients were positioned supine or laterally on a radiolucent table under continuous general or brachial plexus block anesthesia. The operative incisions were performed in accordance with previous surgical incisions when possible. With the sufficient exposure of the infectious site or complete removal of hardware, the devitalized or infected bone and soft tissue were radically resected. The indication of vital osseous tissue was cortical bleeding, which was described as the so-called paprika sign [29]. At least 6 suspected tissues taken from multiple sites were sent for culture in all patients for the postoperative antibiotics. Sufficient irrigation with hydrogen peroxide, iodine liquid, and physiological saline during and after debridement is essential.

**Fig. 1:**
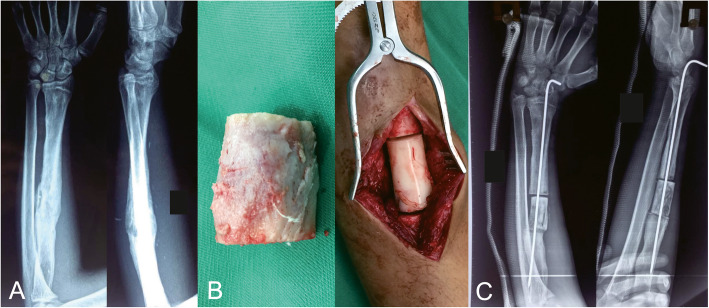
A 25-year-old man who suffered osteomyelitis in his left radius and treated by bone transport technique. **a** Preoperative AP and lateral views of X-rays. **b** Debridement of the infected site with a 4 cm bone defect in left radius and antibiotic bone cement was used to occupy the residual space. **c** Postoperative AP and lateral views of x-rays, the forearm was fixated by Kirschner wire and plaster cast

Antibiotic-impregnated cement spacer was used for the stability of the injured bone. The incision was closed with drainage tubes or vacuum sealing drainage (VSD) for which could not be closed at the initial management or there was a severe active infection. Intramedullary Kirschner wire and plaster cast were used to align the bone ends and preserve the length and orientation of the forearm (Fig. 1)

**Fig. 2:**
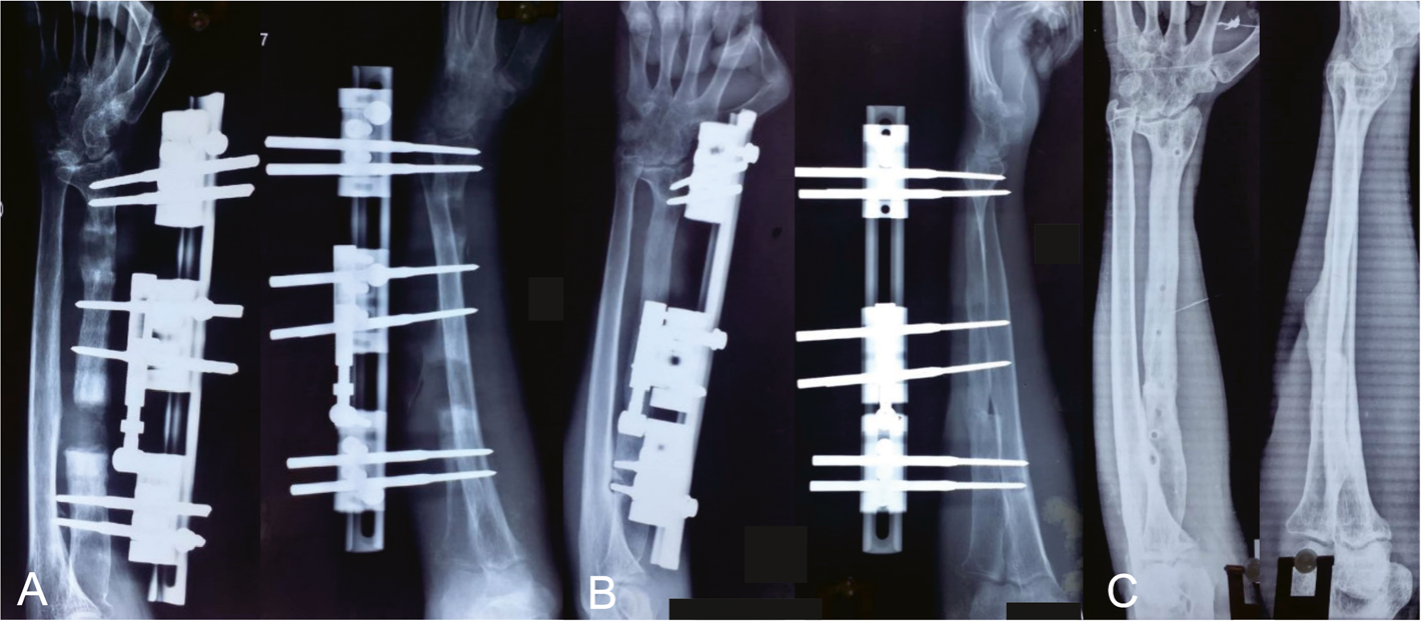
X-rays of the bone transport procedure. **a** One month after the operation with bone transport. **b** Eight months after the operation with bone transport, consolidation of the regenerate and union at the docking site was shown. **c** AP and lateral views of X-rays after removing the external fixator

.

Antibiotic therapy was based on the results of organisms cultures and antibiotic susceptibility tests. The patients were treated with intravenous antibiotics selected by an infectious disease specialist for at least 3 weeks or until the infective process had resolved on the basis of clinical manifestations and laboratory indicators.

#### Stage II(application of monolateral external fixator and osteotomy)

The antibiotic cement spacer and Kirschner wire were removed subsequently after the resolution of active infection. The small wound was managed by local tissue flap or direct suture without tension, while the larger soft tissue defects were resolved by flap transfer or free skin grafting. The standard radiographs were used to plan the placement of the monolateral external fixator.

The length and alignment of the injured bone were restored firstly reference to the contralateral uninjured side. In the neutral position of the forearm and the elbow positioned at 90°, the injured bone was fixated by a monolateral external fixator. Under image intensifier control, two or three hydroxyapatite-coated Schanz screws were inserted on each planned bony fragment, ensuring that every pin was on the same coronal plane. A percutaneous minimally invasive cortical osteotomy was performed at the appropriate site using Gigli saw technique, noticing to preserve the periosteum as much as possible. All the procedures were performed by the same surgical team.

### Postoperative management

All the patients underwent physiotherapy on the second day after surgery, including isometric muscle motion and range of motion exercise for the elbow and wrist. Pushing the wall as stress simulation was an ideal option. Pin site care was performed by medical alcohol or iodophors every day.

Based on the published data [30, 31], bone transport started at a rate of 1 mm daily (4 times a day) following a latency of 7 to 10 days, and the rate was regulated by the specifics of the patients. All patients were required to stay in the hospital at least one week after osteotomy to learn about the pin track care and the regulation of the external fixation for bone transport. To compress the docking site, the bone transport technique was continued for 4 or 5 days after docking.

Regular follow-up was conducted twice a month in the bone transport period, while monthly in the consolidation phase. The external fixator was removed when excellent consolidation and docking site union was achieved (dense bone formation, and corticalization in 3 of 4 cortices on the radiographs). Furthermore, a functional brace was applied for 4–6 weeks. Follow-up was conducted for at least two years after the external fixator removal. The functional results were evaluated by the questionnaire of Disability of Arm, Shoulder and Hand (DASH) and the modified Mayo wrist score (MWS) at the final follow-up.

### Statistical analysis

Statistical analysis was performed with the SPSS 22.0(IBM Corp, USA). Continuous variables were analyzed by paired-samples T-tests and expressed as the mean and range. Statistically significant difference was set at *P* < 0.05.

## Results

**Fig. 3:**
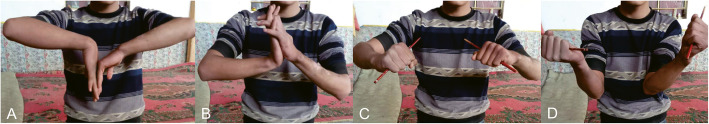
Functional assessment 24 months later, after removing the external fixator. **a** Wrist joint flexion was 20°.**b** Wrist joint extension was 25°.**c** Forearm pronation was 65°.**d** Forearm supination was 25°

The clinical results are shown in Tables 2 and 3. All patients were successfully followed up with a mean period of 28.2 months (24 to 36 months) and achieved an infection-free union (Figs. 1, 2 and 3). There was no recurrence of infection observed. The mean external fixation time was 232.6 days (182 to 276 days), and the mean external fixation index was 46.3 days/cm (40.9 to 61.8 days/cm). The wound healed by themselves, and no patient required flap coverage.

**Table 2:** Details of the treatment outcomes

Case	EFT (days)	EFI (days/cm)	WF/WE(°)		EE/EF(°)		FP/FS(°)		Follow- up(months)
			Pre-op	Follow-up	Pre-op	Follow-up	Pre-op	Follow-up	
1	232	46.4	35-0-40	45-0-45	0-0-110	5-0-125	40-0-20	55-0-35	28
2	247	61.8	30-0-40	50-0-55	0-0-125	10-0-130	40-0-25	55-0-40	24
3	196	43.6	40-0-45	50-0-60	-10-0-115	0-0-125	45-0-30	60-0-45	30
4	253	56.2	20-0-20	20-0-25	5-0-110	5-0-125	35-0-25	50-0-25	24
5	182	45.5	45-0-40	45-0-50	10-0-130	10-0-130	50-0-40	65-0-50	36
6	223	44.6	55-0-50	55-0-50	0-0-125	0-0-125	70-0-65	70-0-65	24
7	184	40.9	40-0-40	50-0-45	0-0-125	5-0-140	40-0-30	50-0-45	32
8	251	41.8	45-0-30	65-0-55	-5-0-110	0-0-145	60-0-55	75-0-65	26
9	239	43.5	30-0-25	45-0-40	0-0-115	5-0-125	35-0-35	50-0-45	24
10	276	42.5	40-0-40	40-0-40	0-0-110	0-0-125	40-0-45	50-0-50	32
11	259	43.1	45-0-45	55-0-50	0-0-120	5-0-135	35-0-40	70-0-55	30
12	249	45.3	45-0-45	60-0-45	5-0-130	5-0-135	45-0-30	60-0-45	28

*EFT* external fixation time, *EFI* external fixation index, *WF* wrist flexion, *WE* wrist extension, *EF* elbow flexion, *EE* elbow extension, *FP* forearm pronation, *FS* forearm supination

**Table 3:** Functional results

Case	DASH		MWS	
	Pre-op	Follow-up	Pre-op	Follow-up
1	34	11	55	85
2	18	5	75	90
3	31	18	70	85
4	49	26	50	65
5	25	10	70	90
6	22	13	80	85
7	26	16	70	80
8	29	12	75	85
9	37	15	60	85
10	30	17	75	80
11	34	12	75	85
12	32	10	70	90
Mean	30.6	13.8	68.8	83.8
t	10.955		-6.760	
*P*-value	*P* < 0.001		*P* < 0.001	

*DASH* the questionnaire of Disability of Arm, Shoulder and Hand; *MWS* the modified Mayo wrist score

The average degree of wrist flexion improved from 39.2° (20 to 55°) pre-operatively to 48.3° (20 to 65°) at the final follow-up, wrist extension from 38.3° (20 to 50°) to 46.7° (25 to 60°), elbow flexion from 118.8° (110 to 130°) to 130° (125° to 145°), elbow extension from 0.4° (-10 to 10°) to 4.2° (0° to 10°), pronation from 44.6° (35° to 60°) to 59.2° (50 to 75°) and supination from 36.7° (20 to 65°) to 47.1° (25 to 65°).

The mean DASH score was 30.6(18 to 49) preoperative, while 13.8 (5 to 26) at the final follow-up. The average modified Mayo wrist score improved from 68.8 (55 to 80) pre-operatively to 83.8 (65 to 90) at the final follow-up. All the differences between the preoperative and final scores were statistically significant (*p* < 0.05). Almost all the patients achieved satisfactory clinical outcomes and were able to perform activities of daily living. The grip strength was markedly improved, and few patients feel pain.

## Complications

Oral analgesics was required for the complaint of pain during the transport period in almost all patients. The most common complication was pin site infection (66.67%), which was resolved by daily pin site care and oral antibiotics. Deep pin tract infection and pin loosening were observed in one patient (8.33%), while pin replacement and intravenous antibiotics contributed to satisfactory outcomes. Axial deviation occurred in one patient (8.33%) and was successfully treated by an open reduction. One patient (8.33%) suffered soft tissue incarceration, procedures including freshening the bone ends, reopening the medullary canal, and excising the invaginated soft-tissue were taken to work; any malalignment was corrected simultaneously. Delayed union of the docking site occurred in two patients (16.67%) and successfully managed by the “accordion” technique. Nonunion was noted in one case (8.33%), while the union was achieved by autologous ipsilateral iliac grafting finally. None of neurovascular injury or psychological problems were observed.

## Discussion

The forearm is a complex joint mechanism and not just two separate bones. The integrity of the anatomical structures is vital for a comprehensive function and synergistic effect, especially for the pronation and supination [28]. There is no doubt that the management of a segmental diaphyseal defect in the forearm is a difficult task for surgeons, especially combined with deep infection. It is so important to reconstruct the forearm function and control the infection process simultaneously.

Various methods have been proposed to treat bone defects in the forearm, including corticocancellous bone graft, nonvascularized fibular graft, vascularized fibular graft, Masquelet’s induced membrane technique, and bone transport [4–6, 9, 10, 12, 26, 27, 32]. Prasarn et al. [33] conducted iliac crest graft in 12 cases with the average defects measured 2.1 cm in the forearm and achieved union in all patients. Although bone graft is an effective method for bone defects, it is not recommended for the defects exceeds 5 cm due to the risk of resorption [34], nonunion, and fracture of the graft [35]. Vascularized fibular graft has a high success rate, but it is technically demanding and has a potential donor site morbidity. Adani et al. [36] performed vascularized fibular graft in 10 patients with an average forearm bone defect of 8.4 cm and achieved union in 9 of 10 patients. Gore et al. [37] reported there was mild muscle weakness after the partial fibula was removed, and Gonzalez et al. [38] declared that there was a statistical link between valgus and the removal of the partial fibula. The Masquelet technique has the advantage of managing the segmental bone defects and required no advanced skills in microvascular surgery, but the outcome is difficult to predict, especially in post-infective defects [39].

Ilizarov bone transport technique, an effective and minimally invasive method that can preserve the biomechanical microenvironment needed for fracture healing, is the preferable option for the treatment of massive bone defect [12, 13, 20, 21, 24]. As early as 1996, Esser had performed a segmental bone transport on a patient who had a posttraumatic defect of his left forearm [2]. The surgery led to complete bone healing, with the patient resuming his activities as a worker. Subsequently, Smith et al. [3] reported 11 consecutive patients with traumatic forearm bone loss who were treated with Ilizarov ring fixation, and the treatment resulted in ablation of infection, healing of atrophic non-unions with minimal complications, and early extremity use. Qun Zhang et al. [26] performed bone transport to treat 16 infected forearm nonunion and acquired satisfied functional results, concluding that radical debridement is the key step to control bone infection. Tang Liu et al. [27] retrospectively reviewed a consecutive series of 21 patients who were treated for the forearm infected nonunion by bone transport, and they concluded that the technique of bone transport after debridement is a safe, effective, and minimally invasive treatment for forearm infected nonunion. Satisfactory outcomes the two studies above gained, while failed to provide more detailed functional scores at the last clinical visit. Recently, Kliushin et al. [28] also reported a 43 years old man with an infected ulnar defect and dislocated radial head due to infected Monteggia fracture, and the patient was successfully treated by Ilizarov bone transport after failed attempts by a bone spacer and fibular graft.

Although the bone transport technique has been used widely, inevitable difficulties as complications that may affect the procedure have been reported by many studies [14, 24, 40]. Pain and pin site infection are common in our study as expected. The other complications, as pin loosening, axial deviation, soft tissue incarceration, delayed union, and nonunion, are also observed. They are all successfully treated with kinds of methods. The key factors we realized to prevent or minimize complications are particular attention, patient compliance, and the surgeon’s experience. Furthermore, our clinical experience has shown that the volume of antibiotic bone cement should be larger than that of the resected bony fragment to create a large enough space, so as to reduce the risk of soft tissue incarceration during the procedure of bone transport.

In the present study, the external fixation index (mean 46.2 days/cm, range 40.9 to 61.8 days/cm) was higher than that in previous study [13, 24, 26, 27] (mean 45.4 days/cm, range 42 to 48.9 days/cm). We considered that the mechanism of bone defect in our study was responsible for this matter. The radical debridement leads to the destruction of the microenvironment for bone regeneration, both docking union and regenerate maturation thereby underwent an excessive duration. Although the range of motion of the wrist, elbow, and forearm are not back to normal, especially for the pronation and supination in the forearm, the functional results were satisfactory with a mean DASH score of 13.8 (5 to 26) and a mean modified Mayo wrist score 83.8 (65 to 90) in our study. There is only a moderate disability in activities of daily life. To recover the forearm function in our experience, intensive physiotherapy should be emphasized during the whole procedure, including the range of motion exercises for the fingers, wrist and elbow within the tolerance of pain from the second day after the operation. The patients were also encouraged to train the affected limbs by pushing the wall as stress simulation [26, 27].

Bone transport can be performed by different types of external fixation. The circular external fixator is not conducive to the functional exercise of the forearm, and the restriction of pronation and supination can exacerbate the functional impairment. Therefore, a monolateral external fixation system without tensioned transfixion wires was used in our study. It is beneficial to the rotation of the forearm, and there is less risk of neurovascular damage [41]. A large amount of movement in the forearm may lead to the loosening of the pins. We recommend using hydroxyapatite-coated Schanz screws to prevent the complications, especially for the patient with osteoporosis. For proper bone transport technique, the most crucial procedure is that the length and alignment of the injured bone must be restored firstly with reference to the contralateral same bone. Besides, the injured bone should be fixated at the neutral position of the forearm and the elbow positioned at 90° in our experience due to the interosseous membrane is most relaxed in this position, and it can reduce the risk of displacement caused by muscle tension.

When conducting an osteotomy on the injured bone, we recommend the use of the Gigli Saw technique. The periosteum may have less regenerative and reparative potential, and the injury to the periosteum may lead to ischemia or necrosis of the underlying bone ends. The subperiosteal Gigli saw osteotomy technique is especially advantageous with bone defect cases to preserves the periosteum while completely transecting the endosteum and eliminates the possibility of an incomplete corticotomy with minimal soft tissue dissection [42].

Our study described an effective alternative technique for the management of bone defects caused by an infection in the forearm. The consecutive stages contain eradication of infection, restoration of bone defect, proper length regain of the injured bone, union achieved and better functions of the wrist and elbow obtained. The most crucial step is radical debridement of the infectious tissues as a priority to establish mechanical stability of the bony fragments and biological stimulation of the bone in our experience. The procedure is lengthy, with a considerable risk of complications. Appropriate insertion of pins, stability of the transport system, meticulous care, and careful attention contribute to ensuring satisfactory results.

The present study had several limitations. Longer follow-up is necessary to evaluate the clinical efficacy better. The absence of a control group and the relatively small sample size required a conservative attitude when interpreting the outcomes of this study. Multi-centered trials with a larger sample size should be conducted in further investigations.

## Conclusions

Ilizarov segmental bone transport technique is an alternative and effective method for the treatment of diaphyseal forearm bone defects caused by infection, and this method acquired satisfactory clinical outcomes.

## Data Availability

The datasets analyzed during the current study are available from the corresponding author on reasonable request.

## Abbreviations

DASH: The questionnaire of Disability of Arm, Shoulder and Hand
MWS: The modified Mayo wrist score
VSD: Vacuum sealing drainage
ESR: Erythrocyte sedimentation rate
CRP: C-reactive protein
